# Revision total knee replacement finances: a detailed cost-analysis of operative practice at a regional tertiary referral centre

**DOI:** 10.1186/s12913-023-10316-x

**Published:** 2024-01-04

**Authors:** Aris Alexiadis, Patrick Reynolds, Louay Al-Mouazzen, Andrew Toms, John Phillips, Ben Waterson

**Affiliations:** 1 Royal Devon University Healthcare NHS Foundation Trust (Wonford), Barrack Road, Exeter, EX2 5DW UK; 2 University of Exeter Medical School, University of Exeter, Exeter, EX1 2HZ UK

**Keywords:** Revision, Knee arthroplasty, RKCC, GIRFT, HRG

## Abstract

**Background:**

The revision knee complexity classification (RKCC) stratifies knee revision operations depending on their level of complexity from simple revisions (R1) to highly complex cases (R3). Current financial codes used for calculation of reimbursement for knee revision services provided at the Trust, rely on patients’ comorbidities. However, previous research has demonstrated that this approach may not yield an accurate financial account of knee revision arthroplasty cost. This is a single centre study from a secondary and tertiary revision unit, with work previously presented by the authors demonstrating that the majority of complex revision knee replacement within the region, take place in this unit. The aims of this study were to illustrate the current cost profile and renumeration service currently in place for revision knee and show the differences in cost based on complexity of the operation.

**Methods:**

In this retrospective study, 90 cases who underwent revision knee operations in 2019 were analysed. Data was obtained from a tertiary referral centre where the episodes had occurred. Mean cost, tariff, and subsequent deficit were calculated for the R1, R2 and R3 episodes.

**Results:**

R2 and R3 episodes were significantly more expensive than R1 episodes. The increase in cost between R3 and R2 episodes was not significant. The total cost of the revision operations was £1,162,343. Tariffs received for R2 and R3 revision operations were significantly more expensive than R1 operations. However, the increase in tariffs received for R3 operations was not significant in relation to R2 operations. The total amount of tariffs received by the Trust was £ 770,996 generating a net deficit of - £ 391,347.

**Conclusion:**

Current financial coding for revision knee does not accurately predict costs associated with revision knee surgery. Net deficit varies depending on the RKCC grade of the knee revision episode with more complex operations resulting in a higher mean net deficit. Implementation of the RKCC could prove to be a useful tool in generating an accurate prediction of the cost associated with knee revision surgery.

**Supplementary Information:**

The online version contains supplementary material available at 10.1186/s12913-023-10316-x.

## Background

Knee arthroplasty is a highly effective surgical intervention with primary total knee replacement (TKR) being one of the most cost-effective interventions offered within a state provided health framework; costing approximately £5,623 per quality-adjusted life year (QALY) [1]. However, knee arthroplasty is a resource-heavy intervention with 62,063 being performed in 2018/2019 alone in the UK [2]. In addition, cost-effectiveness greatly varies depending on factors such as patient comorbidities and where the patient receives their treatment amongst numerous other factors [3]. This is further complicated by the need for revision knee surgery which is required in approximately 6% of all knee replacement’s (KR) performed in the UK [2]. This percentage has remained stable throughout the past few years [2]. Not only is revision surgery more technically complicated, but the risk of post-operative complications and infection is much higher than in a standard KR [2, 4]. This can lead to revision surgery costing up to three times more than the original KR [5].

The Get It Right First Time (GIRFT) [2] initiative was introduced to evaluate and encourage structural changes within NHS trusts and regions with patient outcomes being improved upon while also maximizing cost-effectiveness. The revision knee complexity classification (RKCC), proposed by a group of UK-based orthopaedic surgeons, sought to incorporate the GIRFT philosophy and apply it to revision knee services [6]. The RKCC seeks to classify revision knee surgeries into three categories based on factors such as complexity, patient comorbidities, and previous surgery including other technical factors [6]. Additionally, the RKCC enables multidisciplinary discussion amongst networks, so that patients end up receiving a network considered approach to their surgery.

It has previously been found that centres that have a higher volume of knee revision cases *per annum* are associated with improved patient outcomes and lower complication rates bringing down the cost of patient management [7].

The authors have previously discussed cost complexity of revision total knee replacement [8]. The care episode cost is most likely to be driven by the complexity of the revision procedure; more complex cases being associated with increased theatre time, increased blood loss, increased risk of patient morbidity, longer length of stay in hospital. The authors have also discussed how remuneration for revision knee replacement currently is not reflective of complexity but suggest that this potentially ought to be the case [8]. This was shown to be the case by Petrie et al. (2021) at another high-volume centre, whereby it was demonstrated that the current tariff and remuneration scheme for RKR service results in units recording losses for almost all procedures carried out and places an increased financial burden on units carrying out higher volumes of complex work [9].

Our aim in this study was to analyse the difference in cost among knee revision surgeries of varying complexity performed at a tertiary referral centre, the Royal Devon University Healthcare NHS Foundation Trust. These costs can act as a bench mark for discussions across regions and networks as well as providing a framework for funding to follow complexity and activity using the RKCC as a clinical tool to stratify complexity of revision knee surgery.

Aims:

1. Detail the cost profile and tariff remuneration profile for RKR in a single high-volume centre.
2. Detail the financial burden of performing RKR surgery by complexity, as determined by a validated tool (the RKCC).
3. Demonstrate through modelled analysis the financial burden of increasing complex workload on a high-volume tertiary referral revision centre.

## Methods

### Patient inclusion

Patients undergoing RKR with care episodes dated between 01/01/2019 and 31/12/2019 were included in this study. Only procedures carried out at the Royal Devon University Healthcare NHS Foundation Trust were considered.

### Revision complexity

Each patient undergoing RKR went through the departmental multi-disciplinary meeting (MDT) and the complexity of their proposed procedure was determined (R1, R2 or R3) using the Revision Knee Complexity Classification (RKCC) checklist [6].

### Financial data

For each patient the hospital financial department was able to provide a breakdown of care episode cost, tariff and relevant Healthcare Resource Group (HRG) codes that contributed to the tariff received. Mean care episode cost and mean tariff received for each revision complexity level (R1, R2 and R3) was calculated.

### Outcome measures

The primary outcome measure was the care episode cost. Secondary outcome measures were the procedure tariff and the financial deficit (positive or negative), calculated as the difference between care episode cost and tariff received.

### Financial projections

The mean tariff, mean cost and mean financial deficit for each revision category (R1, R2, R3) was calculated. These values were used to look at the effect of redistributing regional complexity. A calculation was carried out to look at the hypothetical additional spend of carrying out only R2s and R3s within this revision centre, using identical yearly caseload and with similar breakdown of R2 and R3 proportion.

### Tariff

The Tariff represents the remuneration award to a trust for provision of a particular procedure or service once the coding process from the care episode has been completed. The total tariff represents a calculation based on numerous factors; procedure carried out, length of stay, patient comorbidities and a market forces factor which is applied regionally. Clinical coding is used to calculate the tariff from inpatient documentation. This is achieved through the use of the aforementioned HRG codes. The tariff for episode cost for a given procedure or revision complexity level (R1, R2 and R3) will therefore be within a range rather than an absolute value, and the reason mean tariffs were used for any financial estimates.

### Deficit

The sum of the tariff subtracted by the cost of a episode. In this case, an episode of a revision knee surgery. Additionally, the mean tariff is an average of the aforementioned sums.

### Statistical analysis

Data are expressed as mean ± standard deviation. Unpaired t-tests were performed assuming unequal variance to determine statistical significance. No more than two groups were statistically compared in the current study at a given time. Results were considered statistically significant with a *p* value ≤ 0.05. Statistical analysis was performed using Microsoft Excel software 2020.

## Results

### Surgeon volume

The 90 operations analysed in this retrospective study were performed by four surgeons as shown in Table 1 below.

**Table 1:** Revision surgery volume (01/01/2019–31/12/2019)

Volume (number of patients)	Number of surgeons
0–9	1
10–19	1
20–40	2
Total	4

Mean surgeon volume was 22.5 operations per surgeon demonstrating a high volume of revisions per surgeon. Table 2 below displays the demographic details of the patients according to the RKCC classification of their episode of care.

**Table 2:** Demographic details of patients undergoing revision knee surgery

	R1	R2	R3
N. of episodes of care	49	28	13
Mean age (years +/- SD)	72.45 (+/- 14.34)	75.14 (+/- 11.76)	71.23 (+/- 7.30)
Sex	Male:17 Female: 32	Male: 17 Female: 11	Male: 5 Female: 8
Mean BMI (+/- SD)	32.01 (+/- 5.52)	33.11 (+/- 7.76)	32.25 (+/- 5.47)

A HRG code is the output of the clinical coding process, and is determined by the diagnostic codes, operative procedure codes, and comorbidity and complication codes. A range of HRG codes with an associated tariff was generated for the procedures included in this study (R1: £2,176.53 - £ 24,718.66, R2:£ 2,955.87 - £24,219.85, R3:£ 7,844.73 - £26,821.46) This highlights the range of tariffs inked to RKR and demonstrates how the revision complexity level doesn’t contribute to tariff.

### Balance

**Fig. 1:**
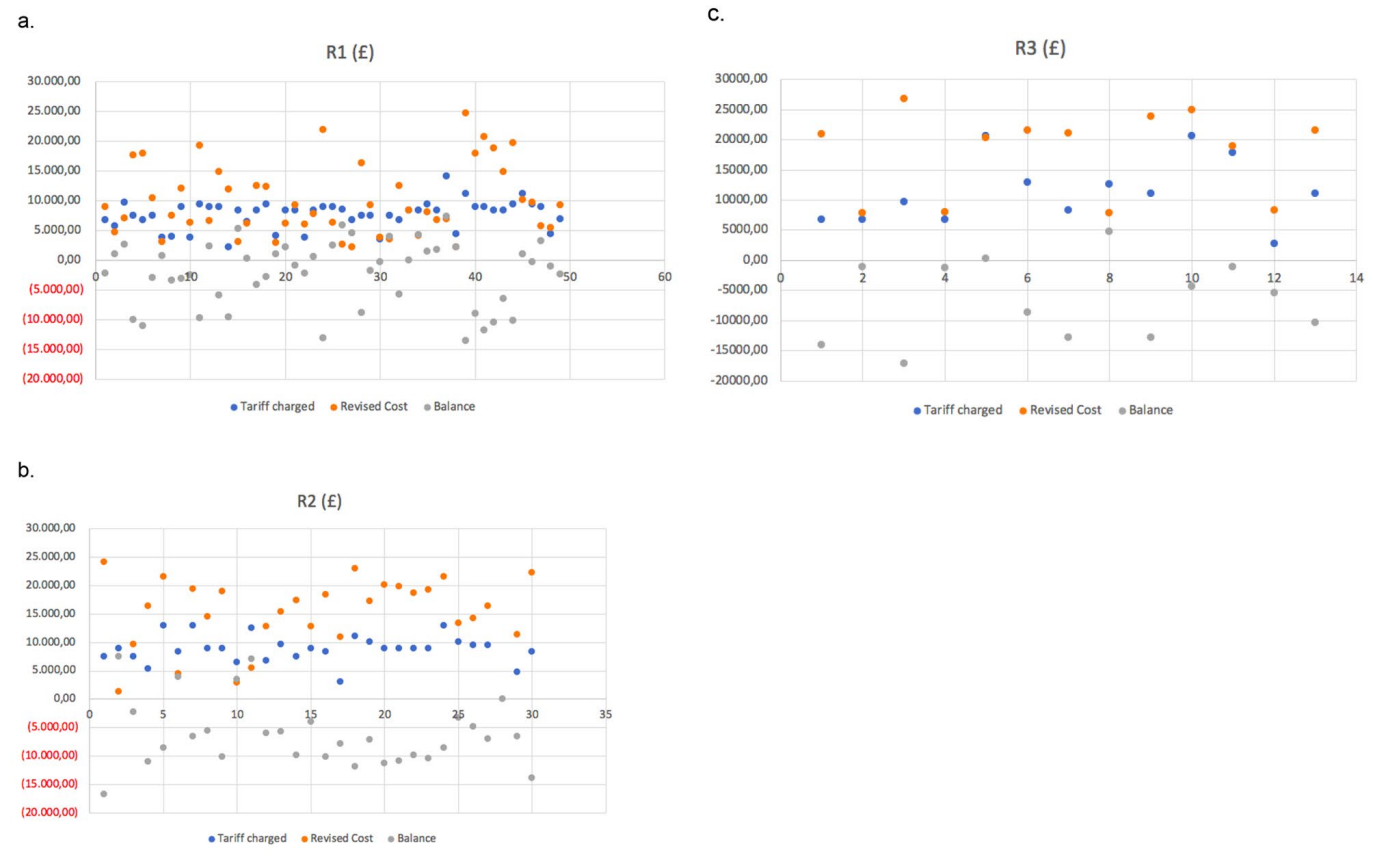
Graphical display of variation in revision cost and tariff reimbursement based on RKCC classification. (**a**) R1 episodes of care; (**b**) R2 episodes of care; (**c**) R3 episodes of care

As previously shown, there was a noticeable discrepancy in cost of a knee revision episode and the tariff received by the RD&E for performing revision knee operations and episode of care. In Fig. 1, the revision costs and tariffs were graphically displayed on a dot plot to better visualise this difference along with the net balance generated from these episodes of care.

Below are examples of the different indications for the different revision knee operations performed. Additionally, many of these indications overlap or co-exist in patients (Table 3 below). It is for this reason that the role of MDT is heavily emphasised within the RKCC guidelines in order to appropriately categorise knee revision operations and their subsequent care.

**Table 3:** Indications for revision surgery analysed in this retrospective study

Indication for knee revision surgery
Infection
Wear of polyethylene component
Pain
Aseptic loosening
Bone loss
Progression of osteoarthritis
Recurrent dislocation
Peri-prosthetic osteolysis
Poor extension

### Cost of operations & tariffs received

The mean cost of each episode of care was calculated and organised based on their RKCC classification. This was done in order to observe whether an increase in operational complexity and presence of patient comorbidities would translate into a greater financial cost to the Trust and if so, by how much (Table 4). In addition to cost of care, tariffs received for undertaking the patients included in this study were also analysed to determine differences in reimbursement based on RKCC category (Table 4).

**Table 4:** Mean revision cost and tariff reimbursement for episodes of care by RKCC classification

RKCC Score	Mean care episode cost (£ +/- SD)	Total Revision Cost (£)	*p*-value	Mean Tariff (£ +/- SD)	Total Tariff (£)	*p*-value
1 (n = 49)	9,9365.59 (+/- 5,894.72)	486,892.81	*p* < 0.000048 (R1 vs. R2)	7,646.50 (+/- 2,304.73)	374,678.71	***p *** ** < 0. (R1 vs. R2)**
2 (n = 28)	15,774.31 (+/- 5642.06)	425,906.42	***p *** ** < 0.38 (R2 vs. R3)**	8,859.94 (+/- 2370.15)	248,078.36	***p *** ** < 0. (R2 vs. R3)**
3 (n = 13)	17,857.24 (+/- 7149.05)	232,144.06	*p* < 0.0019 (R1 vs. R3)	11,402.97 (+/- 5,536.27)	148,238.66	***p *** ** < 0.0 (R1 vs. R3)**

Mean cost for an operation increased with greater RKCC complexity. The costs of R2 (£ 15,774.31 +/- 5,642.06) and R3 (£ 17,857.24 +/- 7149.05) operations were significantly more expensive (*p* < 0,000048; *p* < 0.0019) than R1 operations (9,9365.59 +/- 5,894.72). There was not a significant increase in cost between R2 and R3 operations (*p* < 0.38). The total cost of the revision episodes (n = 90) was £1,162,343.

In a similar manner to cost incurred, the value of tariffs received increased by RKCC complexity. Tariffs received for R2 (£ 8,859.94 +/- 2,370.15) and R3 (£11,402.97 +/- 5,536.27) operations were significantly more expensive (*p* < 0.033; *p* < 0.033) than R1 operations (£ 7,646.50 +/- 2,304.73). While there was an increase in mean tariff received for R3 operations (£ 11,402.97+/- 5,536.27) this was not significant in comparison to tariffs received for R2 operations (£ 8,859.94 +/- 2,370.15) (*p* < 0.13). The total amount of tariffs received by the RD&E amounted to £770,996. This amounted to a net deficit of - £ 391,347 when subtracted from what the Trust spent to provide these operations and episodes of care. There is a discrepancy between the projected costs of a knee revision episode of care and what the actual cost associated with the episode of care is.

### Loan kits

It is not uncommon for complex revision TKR procedures to require specialised implants that are not supplied to a revision unit under contract with industry. These specially sought implants are commonly referred to as “loan kit”. Loan kit was used in six of the 90 episodes of care analysed in this study. Total loan kit costs amounted to £2,259.25 at an average cost of £376.54 per procedure. However, in two of the six episodes of care in which the loan kit was used, specific reduced-cost arrangements were made with the Trust. This is important for considering the implementation of the aims of GIRFT across a regional network; if specific cost-reducing arrangements are in place within a specific revision centre then reducing loan-kit spend may be addressed by redistribution of caseload within the network. The authors have also previously demonstrated that lower volume centres within the region had a higher proportional loan kit usage [10]. It is likely therefore that our regional high-volume centre loan-kit data does not represent the experiences of the region as a whole. Loan costs provided are included in the total costing data provided in Table 5.

**Table 5:** Deficit generated by each RKCC revision surgeries

RKCC Classification	Mean Deficit (Mean Tariff - Mean Cost) (£ +/- SD)	Total Deficit (£)	*p*-value
1 (n = 49)	-2,290.08 (+/- 5482.20)	− 112,214.10	*p* < 0.00049 (R1 vs. R2)
2 (n = 28)	-6,972.41 (+/- 5274.97)	− 195,227.58	*p* < 0.00049 (R2 vs. R3)
3 (n = 13)	-6,454.26 (+/- 6679.22)	− 83,905.40	*p* < 0.054 (R1 vs. R3)

### Deficit

Average deficit was then calculated based on RKCC classification to see whether there would be any difference amongst the RKCC groups in terms of deficit generated.

The mean deficits generated by R2 and R3 episodes, were similar to each other while being much higher than R1 episodes. Interestingly, it was R2 (£ -6,972.41+/- 5274.97) operations that generated the largest mean deficit to the Trust. There was a significant (*p* < 0.00049) increase in deficit between R1 (£ -2,290.08+/- 5482.20) and R2 operations (£ -6,972.41+/- 5274.97). While when R3 (£ -6,454.26+/- 6679.22) operation tariffs were raised to R1 (£ -2,290.08+/- 5482.20) operations, this was not significant (*p* < 0.054). Additionally, there was no significant difference (*p* < 0.81) between R2 (£ -6,972.41+/- 5274.97) and R3 (£ -6,454.26 +/- 6679.22) operation tariffs. The total deficit generated by the 90 operations analysed in this study was £ − 391,347.08.

### Regional projection

In this retrospective study, 49 R1s were included in the financial analysis. We consider the case whereby these are replaced with R2s and R3s in proportionate amounts. In this study, the difference between mean R1 and mean R2 loss was £4,682.33, and the difference in mean R1 and mean R3 loss was £4164.18. We consider the total cost of incorporating these differences into a yearly volume of 105 cases with 73 R2s and 32 R3s. Performing this calculation yields an estimated additional loss of £ 234,605.45.

## Discussion

This retrospective cost-analysis identified that more complex knee revision surgeries, when stratified using the RKCC classification, were associated with a higher mean cost. There was also an increased reimbursement, the more complex an operation was. *However, the average reimbursement was not enough to cover the additional cost.* This led to a greater mean deficit across all RKCC operations with R2 episodes of care being the largest deficit generator. According to data from other Trusts’, the mean deficit increases from R1 to R3 with R3 being the largest deficit generator [8]. Additionally, a large source of cost in revision knee operations is infection [9]. This commonly results in an extended length of stay and antibiotic treatment for patients [9]. It is possible that there are more R2 operations due to infection than R3 operations. Conversely, it could be the case that the current coding process does not accurately account for the degree of complexity and cost seen in an R3 episode. As there were fewer R3s carried out, it may be the case that the lower volume of R3 cases makes the mean more sensitive to outlier effect. For complex reconstructions such as R3s, it is more likely that the use of loan kit is required. Specific financial arrangements were in place in this particular revision centre for the provision of loan kit and as such in these instances the recorded cost may be artificially low, and therefore may not accurately reflect the loan-kit cost burden of other local revision units.

The South West region of the UK is a fitting case study for a cost based analysis, it has a stable network and has been using the RKCC in clinical practice since 2018. In line with GIRFT philosophy and recommendations [2], the South West region formed a preliminary network of care, the South West Knee Group [10]. This network consisted of Trusts across the region who, through the implementation of the RKCC had a platform through which MDT discussion could take place at regional and local level [10]. The creation of such networks has also occurred in other parts of the UK such as in the East Midlands [11].

The operations and remaining episodes of care of this study were performed at a centre which had a surgeon case-load in line with the aims of GIRFT. The lowest volume surgeon (Table 1) was a new consultant who was routinely performing revisions on a dual consultant basis and these cases do not appear on his numbers shown here [7]. Previously it has been demonstrated that centres not reaching the thresholds set out by GIRFT may have worse clinical outcomes which may result in greater financial costs to the Trust in addition to worse outcomes for the patient. It may be reasonable therefore to assume that centres who are not meeting these thresholds could potentially be running greater deficits on their revision knee operations per case. Additionally, lower-volume centres may have to rely more on loan kit, at the risk of further driving up the immediate cost of revision surgery [10]. For example, off-the-shelf equipment such as implants, are significantly more expensive than customised implants [12].

The RKCC provides a simpler system of complexity stratification than the current financial system in Trusts across England and Wales. The current system relies on diagnostic procedural and comorbidity/complication coding to generate the HRG codes that define the tariff rather than the complexity of the operation in question. In knee revision surgery, while important, complications are not the sole contributor behind the cost of surgery. For instance, the presence of infection in revision knee surgery has been previously found to drive up by the average cost of surgery three-fold [5]. HRG coding, as opposed to RKCC stratification, does not take this into account. The RKCC, in addition to its subcategories, looks at patients factors in addition to comorbidities, presence of infection, extensor mechanism compromise and whether there is soft tissue involvement [6].

As seen in this case, use of HRG and RKCC as systems for reimbursement lead to different financial figures. This has been found to be the case in other revision centres in England [8]. A possible reason for the discrepancy is due to the familiarity of the two systems to clinical staff. The financial teams in charge of the financial coding are largely non-clinical staff while clinical staff are not always familiar with the HRG codes and inter-user operability varies from person to person. This can result in a similar operation with a similar patient profile leading to vastly different HRG codes being generated if the surgeon is different [13].

The limitations of using the RKCC to determine tariff are that the checklist does not take procedure complications or duration of care episode into account, and these are key elements of producing a HRG code [14]. Complication risk and care episode length are however likely to relate to complexity, which the RKCC does address. A top-up payment for complex revision KR has been proposed and it may be the case that this will address this problem.

A potential weakness of this study is that the financial data represents a snapshot of revision practice within a relatively short space of time, namely one year. It is likely however that this data reasonably represents routine practice. Additionally, this study does not look at what are the greatest contributors to the cost of a revision knee episode of care (e.g. length of stay, antibiotics). However, previous data has found that length of stay and theatre time significantly increases when comparing R1 and R2 operations [8]. The reason for this is likely to be that R2 procedures cover a broad spectrum of revision TKR indications, including infection, which can involve long and costly procedures and care episodes. In addition, no difference was identified between R2s and R3s compared over the same domains; the reasons for this again may be that R2s cover a broad range of indications for revision TKR with greater cost variability and as such difference was not detected.

The data presented in this study was collected from procedures and care episodes that pre-date cessation of elective activity due to the Covid-19 pandemic. Post-pandemic recovery is one of the current aims of GIRFT [15]. There may be associated financial considerations for revision TKR units going forward that were not a factor when this data was collected. Once recovery progresses and operative caseloads return to pre-pandemic rates, it may be useful to re-examine financial data again to determine if the overall picture has changed, and if there has been any financial legacy effect on the cost of delivering regional TKR service.

In summary, based on the current findings and in line with previous data, further funding is required for the major revision centres to not function at a financial loss. Future studies should seek to break down costs and compare differences in deficits between Trusts. Additionally, as this study is focused primarily on the South West region, future studies could see whether this trend is repeated across other network regions in England & Wales to see the effect of the new networks on the cost of revision knee surgery.

## Conclusion

This paper demonstrates the large discrepancy in cost and reimbursement received for performing a revision knee operation at an NHS England Trust. On average, the deficit for RKRs depended on their RKCC classification: R1 £ 2,300, R2 £ 7,000 and £ 6,500 for R3 operations. Additionally, it shows that the RKCC not only can provide a robust model for revision knee operation stratification through inter-centre MDT dialogue, but also could provide a new financial model through which the true cost of revision knee surgery could be obtained, and guide decision-making in terms of extent of any top-up payment received.

### Electronic supplementary material

Below is the link to the electronic supplementary material.

Supplementary Material 1

## Data Availability

The data that support the findings of this study are available from Royal Devon University Healthcare NHS Foundation Trust, but restrictions apply to availability of these data, which were used under licence for the current study, and so are not publicly available. Data are however available from the authors upon reasonable request and with permission of Royal Devon University Healthcare NHS Foundation Trust. Please contact Andrew Toms in the case of a request for the raw data.
