# Supplementary material for: Revision total knee replacement finances: a detailed cost-analysis of operative practice at a regional tertiary referral centre
Source: BMC Health Serv Res. 2024 Jan 4;24:19. doi: 10.1186/s12913-023-10316-x (PMC10768223; doi:10.1186/s12913-023-10316-x)
Supplement: Supplementary file 1 — Supplementary Material 1 [file 12913_2023_10316_MOESM1_ESM.docx]

**Supplementary Material**

Revision knee complexity classification (RKCC)

| **R1 (Revision 1) - less complex revision surgery**  Examples:  Primary/unicompartmental UKA - aseptic loosening, simple instability, revision of partial to TKA, or polyethylene exchange  AOR 1 or 2A bone loss (no requirement for supplemental metaphyseal fixation)  Debridement with implant retention (DAIR) for acute infection  No significant confounding factors or PIES (co-morbidities, infection, extensor or soft-tissue compromise) |
| --- |
| **R2 (Revision 2) - complex revision surgery**  Examples:  AORI 2B - bone loss requiring metaphyseal fixation e.g. cones or sleeves  Re-revision operations  Stiff knees for revision that may require enhanced exposure techniques such as tubercle osteotomy  Revision for first-time infection  Revision for femoral periprosthetic fracture around primary implant  Complex instability - where correction of the joint line to achieve stability may require the use of cones or sleeves with/without large augments  Includes R1 cases with significant confounding factors or PIES (co-morbidites, infection, extensor or soft-tissue compromise) |
| **R3 (Revision 3) - most complex and salvage cases**  Examples:  Multiple previous revisions  AORI 3 - balance of massive prosthesis +/- metaphyseal reconstruction  Requires hinge for massive bone loss +/- ligament instability  Revision for periprosthetic fracture around stemmed implant or non-union  Recurrent infection after previous revision surgery  Consideration for salvage: arthrodesis, amputation or suppression therapy |
